# Editorial: The role of mitochondria and ferroptosis in cell fate

**DOI:** 10.3389/fcell.2022.1025709

**Published:** 2022-11-25

**Authors:** Yingwen Lin, Haifeng Zhang, Hao Chen, Siim Pauklin, Qingchun Zeng

**Affiliations:** ^1^ State Key Laboratory of Organ Failure Research, Department of Cardiology, Nanfang Hospital, Southern Medical University, Guangzhou, China; ^2^ Guangdong Provincial Key Laboratory of Shock and Microcirculation, Southern Medical University, Guangzhou, China; ^3^ Bioland Laboratory Guangzhou Regenerative Medicine and Health Guangdong Laboratory, Guangzhou, China; ^4^ Department of Cardiology, Sun Yat-sen Memorial Hospital, Guangzhou, China; ^5^ Department of Gastroenterology and Hepatology, Guangdong Provincial People’s Hospital, Guangdong Academy of Medical Sciences, Guangzhou, China; ^6^ Botnar Research Centre, Nuffield Department of Orthopaedics, Rheumatology and Musculoskeletal Sciences, University of Oxford, Oxford, United Kingdom

**Keywords:** mitochondria, ferroptosis, reactive oxygen species, metabolism, LncRNA, long noncoding RNA

Ferroptosis, a regulated form of cell death driven by iron-dependent lipid peroxidation, has emerged as an important process in broad range of pathological conditions and human diseases (Chen et al.). Classically, mitochondrion has a central role in the regulation of cell death and recently has been the focus of extensive research, involving varied biological processes. (Tang et al.; Zhuo et al.; Xia et al.). Upon induction of mitochondrial apoptosis, mitochondrial membrane permeabilization (MMP) usually commits a cell to die. Considering the central role of mitochondria in oxidative metabolism and apoptotic regulation, there exists an intrinsic interaction between ferroptosis and mitochondrial function. In this Research Topic, accumulating studies explored the role of mitochondria and ferroptosis in controlling cell fate by bonding with other cellular processes and revealed the potential regulatory mechanism between them.

Meihe et al. has established the connection between ferroptosis and NLRP1 inflammasome activation with rat’s oxidative stress model (Hydroxyurea supplement) and H_2_O_2_-induced trophoblastic cell model, providing a novel target for adverse pregnancy. Oxidative stress reflects an imbalance between intracellular reactive oxygen species (ROS) levels and the antioxidant defense systems. This excessive ROS, no matter from mitochondrial ETC or external stimulants, is the major driver of Fenton reaction and polyunsaturated fatty acid (PUFA) peroxidation, leading to initiation and promotion of ferroptosis (Gao et al., 2019). Previous research has related ferroptosis to inflammatory reaction, with concentration on tumors development, cardiovascular diseases, and neurodegenerative diseases (Chen et al., 2015; Chen et al.; Tang et al., 2021). NLRP1 inflammasome affects the occurrence and development of ferroptosis while silencing or activating ferroptosis affects the expression of NLRP1 inflammasome, NLRP3 and pro-inflammatory cytokines.

Duan et al. demonstrated that α1-nAchR-Mediated Signaling is the main driver of nicotine-induced NLRP3 inflammasome activation and nicotine-accelerated atherosclerosis and stressed that lipid raft is the essential platform for the combination of nicotine and α1-nAchR. Lipid raft participated in many cellular processes, which was considered to play an essential role in cell signaling transduction, such as pro-apoptosis and inflammation. NLRP3 inflammasome activation has been considered as a key catalyst for atherogenesis. In this study, silencing of α1-nAChR blocked nicotine-induced caspase-1 activation and IL-1β secretion in macrophages. Moreover, the use of MβCD, a lipid raft destructor, inhibited nicotine-triggered NLRP3 inflammasome activation and reduced nicotine-induced macrophage recruitment to atherosclerotic plaque, thus preventing the progression of atherosclerosis in the aorta of apoE−/− mice fed with high-fat diet.

Tang et al. summarized the effect of iron on mitochondria in glaucomatous injury. Glaucoma is characterized by progressive retinal ganglion cell (RGC) damage. RGCs are energy-intensive neurons that highly depend on mitochondrial homeostasis. The authors summarized the role of metal ions and their associated metalloproteins in maintaining mitochondrial homeostasis and cell fate of RGCs. Iron is crucial for mitochondrial biogenesis, motility, and dynamics in RGCs. Mitochondria require iron for the respiratory chain polynuclear sulfur-bridged iron-sulfur (Fe/S) centers residing in the cristae membrane, which are critical for oxidative phosphorylation and ATP biogenesis.

Guo et al. have previously reviewed the role of Heme in cardiovascular diseases. In brief, the detrimental role of heme has been identified in several cardiovascular diseases, including atherosclerosis, heart failure, myocardial ischemia-reperfusion injury, calcific aortic valve stenosis, possibly through ferroptosis. Excess heme which occurs in sickle cell anemia upregulated Hmox1 and increased free iron, driving cardiomyopathy through ferroptosis. Additionally, this Hmox1-dependent heme degradation and excessive free iron accumulation in mitochondria promotes doxorubicin-induced ferroptosis and heart toxicity (Fang et al., 2019). Accordingly, mitochondria iron metabolism might bridge the gap between iron overload and ferroptosis.

Chen et al. provided a unique review on the emerging role of ferroptosis in liver diseases. They presented detailed information and supporting evidence to indicate that ferroptosis is a noticeable mechanism in the pathogenesis of a wide spectrum of live diseases, including alcoholic liver disease, nonalcoholic fatty liver disease, viral hepatitis, autoimmune hepatitis, fibrosis, hepatocellular carcinoma and others. This current review deepens our understanding the biological characteristics and regulatory mechanism involved in liver diseases and provide new insight into therapeutic approach for liver diseases by targeting ferroptosis. Meanwhile, they urged the searching for accurate *in vivo* biomarkers for ferroptosis and expected clinical studies investigating the application of specific ferroptosis inducers or inhibitors in treating liver diseases.

As a form of autophagy, mitophagy is a key process that determines cell fate and maintain cellular homeostasis by selectively removing dysfunctional mitochondria. Recently, Zhuo et al. established a mitophagy-related gene signature derived from The Cancer Genome Atlas (TCGA) cohort and validated its prognostic value in International Cancer Genome Consortium (ICGC) cohort and two Gene Expression Omnibus (GEO) cohorts. The risk score was shown to independently associate with high-risk pancreatic cancer and poor outcomes. The developed nomogram possessed high predictive value for long-term survival, indicating an important role of mitophagy in pancreatic cancer and provide a novel prognostic model to identify high risk individuals in patients with pancreatic cancer.

Using the hepatocellular carcinoma RNA transcriptome raw count data (LIHC) from The Cancer Genome Atlas database (TCGA, https://portal.gdc.cancer.gov/), Xia et al. identified eight mitochondrion- and ferroptosis-related lncRNAs by Pearson’s analysis, Lasso–Cox regression, and RT-qPCR. Based on these eight lncRNAs, they established the MF-related prognostic signature (LPS), which was shown to have outstanding stratification ability and prognostic prediction capability. Further functional enrichment analysis on the DEGs suggested that these lncRNAs may affect mitochondria functions and ferroptosis in HCC through the cell cycle pathways. This finding shed light on novel regulatory mechanism of mitochondrial function and ferroptosis.

To conclude, various metabolic processes in mitochondria can actively contribute to ferroptosis, determining the cell fate in several ways, which was demonstrated in a broad range of pathological circumstances. Mitochondrion could serve as a regulatory pivot that link ferroptosis with other biological process such as inflammation.
